# Research on cloud manufacturing service recommendation based on graph neural network

**DOI:** 10.1371/journal.pone.0291721

**Published:** 2023-09-26

**Authors:** Minghui Li, Xiaoqiu Shi, Yuqiang Shi, Yong Cai, Xuewen Dong

**Affiliations:** 1 School of Manufacturing Science and Engineering, Southwest University of Science and Technology, Mianyang, China; 2 State Key Laboratory of Digital Manufacturing Equipment and Technology, School of Mechanical Science and Engineering, Huazhong University of Science and Technology, Wuhan, China; Southwest Jiaotong University, CHINA

## Abstract

There are an increasing number of manufacturing service resources appeared on the cloud manufacturing (CMfg) service platform recently, which leads to a serious information overloading problem to the enterprises that need these resources. To tackle this problem, a graph neural network-based recommendation method for CMfg service resources is proposed, which effectively overcomes some limitations of the traditional recommendation methods. Specifically, we first use different similarity calculation methods (e.g., Cosine similarity, Pearson correlation coefficient, etc.) to calculate the similarities between different resources based on the feature information of CMfg service resources. A resource graph dataset is accordingly established. A graph neural network is then used to perform representation learning of nodes in these graphs, obtaining the vector representations of these nodes. Finally, new links that may appear in a graph are predicted by performing dot product calculations on these nodes’ vector representations. And these links can be used to recommend suitable resources. Experiments mainly show that (i) the proposed method obtains better link prediction accuracy compared with that of other link prediction algorithms; (ii) when the network density used for training is relatively high, the predictive performance of the proposed method is improved significantly. Our method can shed light on how to choose suitable CMfg service resources from the CMfg service platform.

## Introduction

Manufacturing industry has shown an obvious trend of service-oriented transformation since the 1980s [1]. It is of great important for manufacturing enterprises to provide value-added services based on their own manufacturing capacity. In this process, service-oriented manufacturing has become an important direction for the transformation and upgrading of manufacturing [2, 3]. Furthermore, with the development of several new generation information technologies (e.g., cloud computing, Internet of Things, big data, etc. [4]), a service-oriented manufacturing model, called cloud manufacturing (CMfg), has emerged [5].

**Fig 1:**
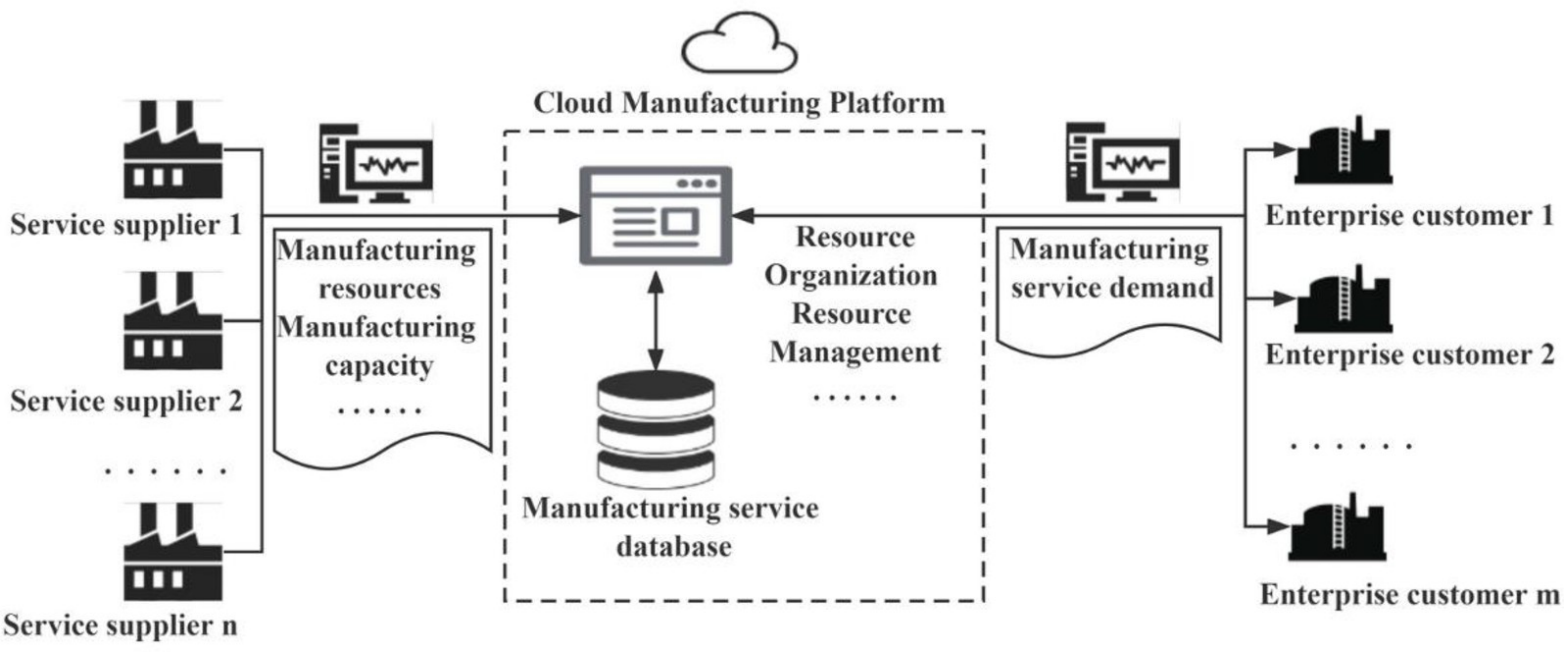
A cloud manufacturing service platform.

In the CMfg environment, enterprises closely connect the massive, heterogeneous, and distributed online manufacturing resources through the encapsulation, release, discovery, and evaluation of manufacturing services, so as to rapidly build a competitive manufacturing service supply chain system, and hence improve the efficiency of resource allocation and the overall competitiveness of enterprises [5, 6]. Fig 1 shows a conceptual diagram of a cloud manufacturing service platform. On one hand, enterprises can integrate and release their idle manufacturing resources on the CMfg service platform according to their own manufacturing situations in order to realize the sharing of manufacturing resources and manufacturing capabilities [7]. Here, manufacturing resources refer to various materials and intangible resources used in the production process, such as equipment, machinery, etc. Manufacturing capability is defined as the production tasks and processing capabilities that a company has within a given time interval, including many aspects such as the production rate of the production line, the availability of equipment, etc. On the other hand, enterprises can obtain suitable manufacturing services on the CMfg service platform according to their own manufacturing service demands, avoiding the difficulties of finding manufacturers offline and reducing the decision-making costs [8]. Therefore, CMfg is becoming a new engine for the transformation and upgrading of enterprises from production-based manufacturing to service-based manufacturing [9].

However, with the increasing scale of global manufacturing industry and the growing number of manufacturing services on the CMfg service platform, the CMfg service system has experienced a serious information overloading problem, and hence business users have encountered greater difficulties in obtaining suitable resources from the huge number of manufacturing service resources [10]. Accordingly, manufacturing service recommendation systems have gained an extensive attention recently [11, 12]. These systems aim at automatically recommending a list of manufacturing service resources that may meet users’ demands [13]. Meanwhile, as an important means of information filtering, the recommendation system is one of the most effective methods to solve the information overloading problem at present [14, 15], which helps to alleviate the serious information overloading problem occurring in CMfg service systems and hence improves the efficiency of CMfg platforms.

Research on the use of personalized recommendation systems for manufacturing service resources has currently yielded partial results, and these studies have mainly been carried out from the perspective of user clustering, resource combination recommendation, and recommendation algorithm optimization [16, 17]. For example, there are some studies that use customer transaction data to obtain the interactions between users and manufacturing service resources, and then use collaborative filtering algorithms to achieve manufacturing service recommendation [18]. In addition, there are also some other studies that use clustering algorithms to cluster and analyse users with different manufacturing service demands from the perspective of user clustering as a way to conduct research on the recommendation of manufacturing service resources [13]. However, traditional recommendation algorithms cannot effectively exploit the hidden features among resources, and can only learn low-dimensional and linear features from past interactions between users and services. They bring high computational costs as the data size increases, leading to unsatisfactory recommendation results [19]. To address this problem, some authors have combined deep learning algorithms with traditional recommendation algorithms (e.g., collaborative filtering (CF)) to improve the accuracy and efficiency of recommendation [9].

However, most of the current research on CMfg service recommendation is based on user clustering or CF recommendation based on users, ignoring the correlation and similarity between manufacturing service resources on the CMfg service platform. To address this problem, a graph neural network-based recommendation method for CMfg services is proposed in this paper, which effectively overcomes the data sparsity and other limitations faced by traditional recommendation methods. The contributions of this study are mainly as follows:

- This study constructs a resource graph dataset based on the similarities of manufacturing service resources, and proposes a CMfg service recommendation method based on graph neural network.
- The impacts of different similarity calculation methods on the proposed method are analysed, and it is found that to a certain extent, a lower link threshold always has a significant improvement on the link prediction performance of the propose method.
- The experimental results indicate that the proposed recommendation method based on graph neural network has achieved higher accuracy in link prediction, and is better than that of other link prediction algorithms.

The rest of this paper is organized as follows. The related work is presented in Section 2. Section 3 focuses on the graph construction work based on real manufacturing service data. Section 4 describes our proposed method and the other algorithms used to compare. Experimental results and analysis are given in Section 5. Conclusions are presented in Section 6.

## Related work

### Cloud manufacturing service platform

CMfg service platform is the key to ensuring the orderly and efficient completion of relevant manufacturing services [20]. It aims to efficiently and dynamically manage the manufacturing service resources released by enterprises on the platform, and can provide suitable manufacturing resources or task solutions to the manufacturing service demand side [21].

Numerous scholars have recently carried out some relevant studies. For example, Huang et al. [22] proposed a CMfg service platform and a manufacturing capability trading platform for small- and medium-sized enterprises (SMEs) in response to some issues exposed in the transformation of traditional manufacturing to service-based manufacturing. They also constructed the corresponding CMfg service platform framework and then discussed the key technologies and features of it. Song et al. [23] constructed a CMfg service platform for SMEs in order to improve the comprehensive competitiveness of them. The authors mainly studied the common engines such as intelligent supply and demand matching engine, logic engine, and credit evaluation engine in the cloud platform. Then, they solved some key problems such as intelligent search, order tracking, and collaborative management of the whole process. In addition, Wei et al. [24] proposed a product platform architecture for CMfg in response to the rapid development of the market. They discussed and analyzed the key technologies involved in the platform construction process. Hasan et al. [25] proposed an improved CMfg service platform architecture framework that enables mass customization services from remote clients and gives some implementation details. Radmanesh et al. [26], after combining with blockchain technology, proposed a CMfg service platform which considers distributed service combinations that can effectively solve the operation management problem in industrial applications. Pan et al. [27] designed a pricing model for CMfg platform based on the bilateral market theory research for the pricing problem under the CMfg model, and by solving the model, the optimal pricing strategy for maximizing the profit of the CMfg platform was obtained.

The aforementioned studies show that the research on CMfg platform has gained an extensive attention and some results have been achieved. However, CMfg platform has an increasing number of manufacturing service resources, which brings a serious information overloading problem to the CMfg service systems. Hence, users encounter greater difficulties in screening suitable service resources from the massive manufacturing services, which affects the efficiency of decision-making [19, 28]. However, the related research on this problem is still lacking, so how to alleviate the information overloading problem of CMfg service platform is the main research topic studied in this paper.

### Recommendation system

Reviewing previous related studies, we find that an effective way to solve information overloading problem is to make personalized recommendation [29, 30]. With the development of information technologies and the Internet, people have gradually moved from the era of information scarcity to the era of information overloading, which greatly affects the efficiency for users of making decisions on various web service platforms [31]. Accordingly, personalized recommendation systems have emerged as an important means of information filtering, which is one of the most effective ways to solve the information overloading problem at present [32].

#### Link prediction

In addition to traditional collaborative filtering recommendation algorithms, link prediction is one of the most widely studied and applied methods in the field of recommendation systems and data mining. Link prediction refers to predicting the possibility of a connection between two nodes in a given network in order to determine whether there is some connection between these two objects [33]. In recommendation systems, social interactions, commodity transactions, and movie recommendations can be modeled as networks, such as social networks, bipartite graph of user items, and item attribute relationship networks, etc. Therefore, link prediction is often used in many recommendation scenarios such as the friend recommendation or movie and product recommendation, obtaining good recommendation results [34, 35].

For example, Yin et al. [36] proposed a deep graph neural network to predict the links on the bipartite graph of user items by using information propagation, and the experimental results showed that the method effectively alleviated the data sparsity problem in the recommendation process and improved the recommendation accuracy. Kaya [37] constructed a user hotel bipartite graph for the hotel recommendation problem. Then, he proposed a hotel recommendation system based on link prediction, achieving good recommendation accuracy larger than 89%. Wang et al. [38] combined similarity structure with a latent feature model and hence proposed a link prediction framework based on bipartite graph, which has better stability compared with that of some existing methods. In addition to the bipartite graph of user items mentioned in the aforementioned study, commodity resources can also be constructed into resource networks based on their existing links to achieve the corresponding resource recommendation tasks.

#### Manufacturing service recommendation

The dynamic and distributed nature of manufacturing service resources [39] determines that the search and recommendation mechanism of CMfg service resources needs more specialized research. How to recommend services in order to meet the needs of resource demanders in large-scale CMfg service resources has become a critical issue in the field of CMfg [40].

For example, Fan et al. [13] conducted service recommendation research from the perspective of manufacturing service clustering, developed a service clustering method by using a Latent Dirichlet Allocation-based topic model to cluster CMfg services into specific domains, and then introduced a domain-aware reputation service recommendation method to recommend highly reputable services in each domain for users. Zhang et al. [41] developed a CF method based on hybrid social networks for recommending personalized manufacturing services. Hao et al. [42] found that most of the traditional recommendation methods ignore the evolutionary characteristics of CMfg service systems, and for this problem, a time-aware target reconfiguration service description method was proposed, leading to a new recommendation strategy for manufacturing services. Li et al. [43] proposed a framework for manufacturing-task semantic modelling and manufacturing-resource recommendation for digital twin shop-floor. In addition, some new techniques such as deep learning and knowledge graphs have led to a new phase of research in the field of recommendation systems [28, 44].

In summary, the current research hotspots in the field of recommendation for CMfg services are data sparsity, cold start problem, and consideration of user preferences in the recommendation process. And with the introduction of deep learning technology, it will bring a further optimization to recommendation systems. However, the current research is less concerned with the similarity and association between manufacturing services. To address the above problems, this study constructs a graph of manufacturing service providers based on their similarities according to the special characteristics of manufacturing services, where each provider corresponds to a node in the graph and each provider can provide a certain kinds of manufacturing services to users. Then, the node representation learning of the graph is obtained, and the training of link prediction model is completed. Finally, based on the results of link prediction, the manufacturing service recommendation for users is achieved.

## Graph construction

### Features and data sets

In order to reflect the characteristics of manufacturing service resources more realistically, this study collects 3,000 manufacturing service resources (items) in MFG.com. MFG.com is one of the most potential cloud manufacturing companies [45]. Behind manufacturing service data is a real existing manufacturing enterprise, and these manufacturing enterprises cover more than 30 kinds of manufacturing services such as casting, 3D printing, assembly, and injection molding. In order to reflect the manufacturing capability information of manufacturing service resources, we use all the manufacturing services involved in the selected 3,000 manufacturing service providers as the feature sets of manufacturing service providers. A total of 34 manufacturing services are selected, so each set contains a total of 34 features. Therefore, each manufacturing service provider corresponds to a feature set, expressed as *M*_*n*_(*n* = 1,2,3,…,3,000). The feature of a manufacturing service provider is expressed as *f*_*i*_(*i* = 1,2,3,…,34). Each manufacturing service provider can be expressed as *M*_*n*_ ={*f*_1_,*f*_2_,*f*_3_,…,*f*_34_}, and further, it can be expressed as a multi-dimensional vector ***M***_*n*_ =(*f*_1_,*f*_2_,*f*_3_,…,*f*_34_).

For each feature in the set, i.e., each manufacturing service, if the manufacturing service supplier has the feature, then *f*_*i*_ = 1, and *f*_*i*_ = 0 otherwise. According to this method, we can get a binary characteristic matrix ***A*** with a scale of 3,000×34. Then, we use the feature vector of manufacturing service providers to calculate the similarity between various suppliers, and build the corresponding adjacency matrix which stores the specific information of a graph. In this paper, we represent a graph as *G*(*V*,*E*), where *V*∈R^*n*×*di*^, R represents the relationship satisfied by the set, *n* represents the number of nodes, *di* represents the dimension of the feature, and *E*∈R^*n×n*^. The nodes in the graph represent manufacturing service providers, while the edges imply the degree of similarity between the corresponding two suppliers. Note that the graph is an undirected graph in this paper.

### Similarity measure

Different similarity measures bring different information about the graph data, so in this paper, a total of four commonly used similarity measures, i.e., Euclidean similarity, Cosine similarity, Pearson correlation coefficient, and Jaccard correlation coefficient, are used to construct the adjacency matrix with different similarity values. The effects of various similarity calculation methods on the link prediction method are also discussed in the paper.

a) Euclidean similarity

Euclidean similarity, also known as Euclidean distance, is the true distance between two points in an n-dimensional space [46, 47]. When measuring the degree of similarity of different vectors, a smaller distance represents a greater similarity between two vectors, and vice versa. The Euclidean similarity of vectors ***a*** and ***b*** is calculated as shown in (1).


$$\mathrm{Euclidean}‐\mathrm{sim}(a,b)=\sqrt{\sum_{i=1}^{n}{\left(a_{i}-b_{i}\right)}^{2}}$$ (1)

b) Cosine similarity

Cosine similarity is a measure of the similarity of different vectors by calculating the cosine value of the angle between two vectors [48]. The cosine value close to 1 (the corresponding angle tends to 0) indicates that the two vectors are very similar, and vice versa. The Cosine similarity of vectors ***a*** and ***b*** is calculated as shown in (2).


$$\mathrm{Cosine}‐\mathrm{sim}(a,b)=\frac{\sum_{i=1}^{n}\left(a_{i}\times b_{i}\right)}{\sqrt{\sum_{i=1}^{n}{\left(a_{i}\right)}^{2}\times }\sqrt{\sum_{i=1}^{n}{\left(b_{i}\right)}^{2}}}$$ (2)

c) Pearson correlation coefficient

Pearson correlation coefficient of two vectors is equal to the product of their covariances divided by their respective standard deviations [49]. The value of the coefficient is always between -1 and 1. Approaching 0 means that the two vectors are uncorrelated, and approaching 1 or -1 means that they are strongly correlated. The Pearson similarity of vectors ***a*** and ***b*** is calculated as shown in (3).


$$\mathrm{Pearson}‐\mathrm{sim}(a,b)=\frac{\sum_{i=1}^{n}\left(a_{i}-\bar{a})(b_{i}-\bar{b}\right)}{\sqrt{\sum_{i=1}^{n}{\left(a_{i}-\bar{a}\right)}^{2}}\sqrt{\sum_{i=1}^{n}{\left(b_{i}-\bar{b}\right)}^{2}}}$$ (3)

d) Jaccard correlation coefficient

Jaccard correlation coefficient is often applied to calculate the correlation of two sets and can also be used to measure the similarity of two binary multidimensional vectors [50, 51]. The Jaccard similarity of vectors ***a*** and ***b*** is calculated as shown in (4).


$$\mathrm{Jaccard}‐\mathrm{sim}(a,b)=\frac{\left|a\cap b\right|}{\left|a\cup b\right|}=\frac{M_{11}}{M_{01}+M_{10}+M_{11}}$$ (4)

*M*_11_ denotes the number of dimensions, where both vectors correspond to dimensional positions, 1. *M*_01_ denotes the number of dimensions, where the dimensional position in vector ***a*** is 0 and the corresponding position in vector ***b*** is 1. *M*_10_ denotes the number of dimensions, where the dimensional position in vector ***a*** is 1 and the corresponding position in vector ***b*** is 0. In the following, the specific values obtained from the four similarity calculation methods are normalized in order to facilitate the unified metric.

### Structure information of the graph

In this section, we bring the manufacturing service resources feature matrix **A** obtained through data processing by using the four similarity calculation methods. We then normalize the data to obtain four adjacency matrices, corresponding to four graph datasets. In order to reduce the computational complexity of the subsequent work, we set a connection threshold between nodes, and the similarity value is set to be 0 when it is lower than the given threshold. The structural information of these graphs is shown in Table 1.

**Table 1:** Structure information of these graphs.

Method	Nodes	Edges	Average Degree	Diameter	Average Path length	Density
Cosine	3,000	2,879,966	1,920.62	3.00	1.36	0.64
Pearson	3,000	2,166,445	1,445.26	4.00	1.56	0.48
Euclidean	3,000	424,890	307.45	18.00	3.66	0.1
Jaccard	3,000	1,498,049	999.70	6.00	1.86	0.33

## Model and results

### Model and algorithm

DeepWalk is one of the most commonly used graph neural network models in the field of recommendation systems [52]. This algorithm is also one of the classical algorithms of network representation learning, and is a common method used to learn the node vector representation in a network. DeepWalk draws on the idea of Word2vec algorithm. Similar to Word2vec, it uses the cooccurrence relationship between nodes in the graph to learn the vector representations of nodes. This algorithm samples nodes in the graph by using random walk, and then learns the embedded representations of nodes by using Skip-Gram.

The basic steps of random walk are as follows. Give an initial node, randomly select a node from the neighbors of this given node with a certain probability, move to this new visited node, and then repeat the above process until *t* nodes are visited. We arrange these visited nodes according to the access order, and then get a sampling sequence of nodes with a length of *t*. Specifically, given a graph *G*(*V*,*E*), select an initial node *v*^(0)^ from the graph, and start walking with it as the initial node. If the node visited in step *t* is *v*^(*t*)^, then the node visited in step *t*+1, *v*^(*t*+1)^, will be selected from *v*^(*t*)^’s neighbors according to the probability given in (5).


$$p(v^{\left(t+1\right)}|v^{\left(t\right)}))=\{\begin{array}{l} 1/d(v^{\left(t\right)}),v^{\left(t+1\right)}\in N(v^{\left(t\right)})\\ 0,\;\mathrm{else} \end{array}$$ (5)

*d*(*v*) represents the degree of node *v*, that is, the number of connected nodes. *N*(*v*) represents the set of neighboring nodes of node *v*. It can be seen that in the DeepWalk algorithm, the next node visited during random walk is randomly selected from the neighbors of the current node according to the uniformly distributed probability. Thus, each node in the graph is taken as the initial node for random walk, and then the sequences obtained from the random walk are brought into the Skip-Gram model to obtain the vector representations of the nodes in the graph. As shown in (6), by calculating the dot product of two vectors, the Sigmoid function is used to obtain the prediction result, i.e., the connection probability *P* between two nodes, so as to recommend the manufacturing service resources to users.


$$P_{\upsilon_{i}∼\upsilon_{j}}=Sigmoid(\phi (\upsilon_{i})\cdot (\upsilon_{j}))$$ (6)

After the node representation learning is completed, the link prediction task in this paper is used to determine whether there is an edge between these two nodes. In this study, we perform the link prediction model as a binary classification problem. First, we take the edges that exist in the graph as positive samples, and on this basis, we negatively sample some edges that do not exist in the graph as negative samples. Then, we divide the positive and negative samples into a training set and a test set, in which the training set accounts for 80% and the test set accounts for 20%. Finally, the score between two node representations is obtained by dot product calculation of node pairs to predict the possibility of the connection between these two nodes.

In addition to the four similarity calculation methods inspired algorithms proposed in this paper, three other link prediction algorithms are used to compare here. Among the algorithms proposed by other scholars, the node similarity-based algorithm has a lower time complexity, while some scholars confirmed that the CN (Common Neighbors) algorithm, AA (Adamic-Adar Index) algorithm, and RA (Resource Allocation) algorithm have a better link prediction performance through network data experiments [53].

CN [54]: The design of this algorithm is based on an intuitive assumption that the higher the number of common neighboring nodes of two nodes, the greater their similarity, defined as (7).


sim(a,b)=|Γ(a)∩Γ(b)| (7)

AA [55]: This algorithm first calculates the set of common neighbors of two nodes, calculates the logarithmic inverse of the degree of each element node in the set, and then sums all the values, defined as (8).


$$\mathrm{sim}(a,b)=\sum_{z\in \Gamma (b)\cap \Gamma (b)}\frac{1}{\log|\Gamma (z)|}$$ (8)

RA [56]: This algorithm first calculates the degree of each common neighboring node of *a* and *b*, calculates their inverse, and finally sums these values, defined as (9).

$$\mathrm{sim}(a,b)=\sum_{z\in \Gamma (a)\cap \Gamma (b)}\frac{1}{\left|\Gamma (z)\right|}$$ (9)

Γ(*a*) represents the neighboring node set of node *a*, Γ(*b*) represents the neighboring node set of node *b*, *sim*(*a*,*b*) represents the similarity between node *a* and node *b*, and Γ(*z*) represents the neighboring set of node *z*.

In addition, some other graph neural network models, i.e., GCN (Graph Convolutional Network) model and GAT (Graph Attention Network) model, are also used to compare. GCN model can effectively convolve the graph data to obtain node representations in the graph. These representations are then used to solve node classification as well as link prediction problems. In this model, the propagation between layers is shown as follows [57].

$$H^{\left(l+1\right)}=\sigma ({\tilde{D}}^{-\frac{1}{2}}\tilde{A}{\tilde{D}}^{-\frac{1}{2}}H^{\left(l\right)}W^{\left(l\right)})$$ (10)

*H*^(*l*)^ represents the feature representation of the *l*th layer, $\tilde{A}$ is the adjacency matrix with added self-connections, $\tilde{D}$ is the degree matrix of $\tilde{A}$, *W*^(*l*)^ is a layer-specific trainable weight matrix, and *σ* denotes an activation function.

GAT model can consider the weigh information of different neighbors when aggregating the neighbor information, and then can weigh the information of these neighbors by the attention score, obtaining new node feature representations. The calculation method for output features is as follows [58].

$${\vec{h}}_{i}^{\prime }=\sigma (\sum_{j\in N_{i}}a_{ij}W{\vec{h}}_{j})$$ (11)

${\vec{h}}_{i}^{\prime }$ represents the output characteristics of node *i*, ${\vec{h}}_{j}$ represents the characteristics of input node *j*, *N*_*i*_ represents the neighboring nodes of node *i*, *a*_*ij*_ is the normalized attention coefficient calculated through an attention mechanism, **W** is a layer-specific trainable weight matrix, and *σ* denotes an activation function.

### Evaluation metrics

In this paper, we use the AUC metric [59] and the Precision metric [60] to measure the accuracy of the link prediction algorithm. The AUC is the probability that the score value obtained by the presence of an edge is higher than the score value obtained by the absence of an edge when chosen randomly in the test set. Among *n* experiments, if there are *n*′times when a link in the test set has a higher score than a non-existent link, and if the two scores of *n*″ times are equal, then the AUC value can be defined as (12).


$$\mathrm{AUC}=\frac{n^{\prime }+0.5n^{\prime \prime }}{n}$$ (12)

AUC measures the accuracy of the algorithm. The higher the AUC value, the more accurate the algorithm.

In addition to using the AUC metric for link prediction evaluation, the Precision metric is also used to measure the accuracy of these algorithms. A higher Precision value indicates a better link prediction performance. Specifically, it is the percentage of the top *L* links with the highest score in the link prediction of the network in terms of the number of correctly predicted links *m*. The Precision can be defined as (13).


$$\mathrm{Precision}=\frac{m}{L}$$ (13)

### Experimental result and evaluation

**Fig 2:**
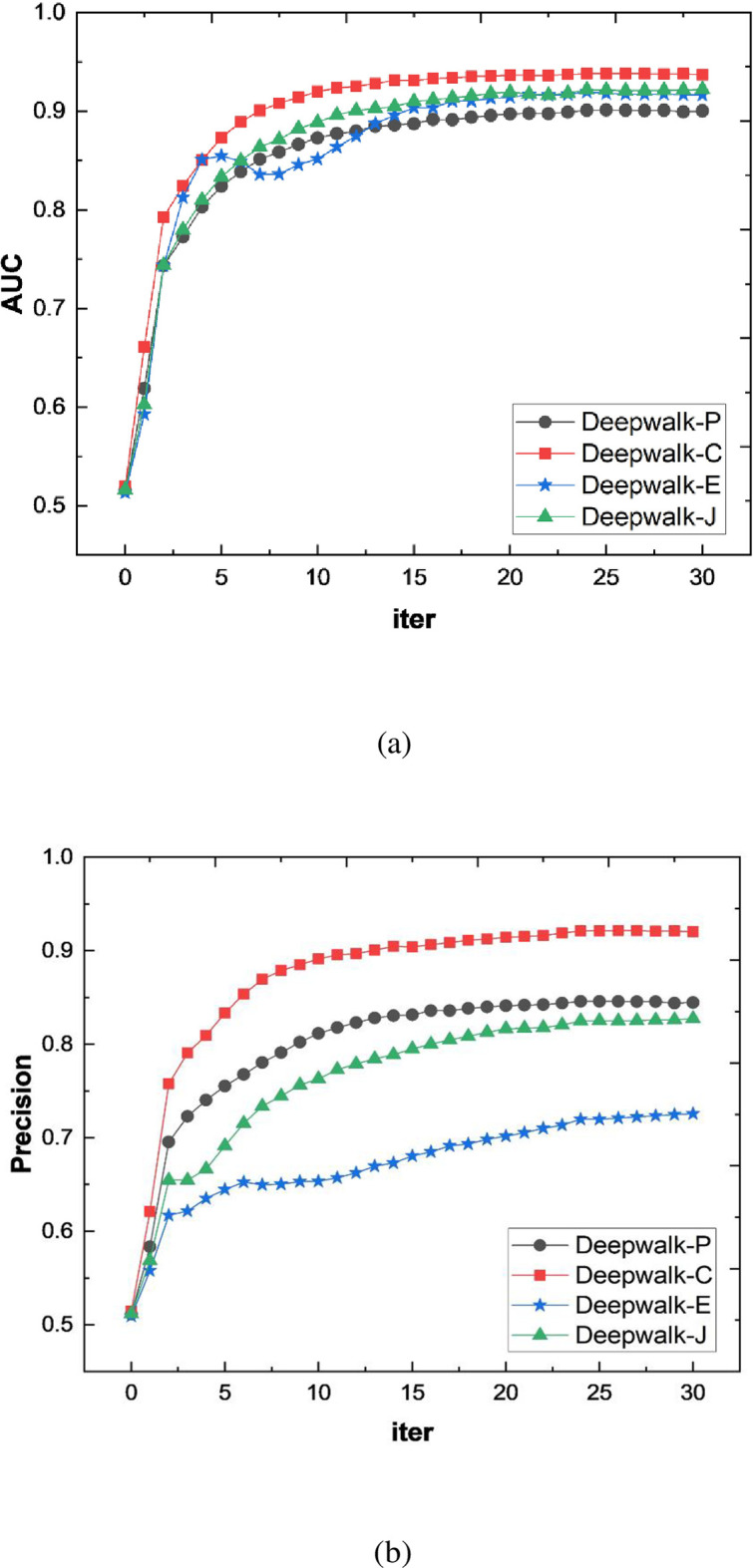
Performance of link prediction model corresponding to different similarity calculation methods.

All experiments are conducted on the open-source platform “PyCharm Community Edition”, and the programs are all written in python language. The training results of the link prediction models are shown in Fig 2. First, we compare the link prediction models based on three similarity calculation methods. Among them, DeepWalk-P means that the link prediction model is conducted on the graph based on Pearson correlation coefficient, DeepWalk-C is based on Cosine similarity, and DeepWalk-E is based on Euclidean similarity. The experimental results show that the link prediction model based on DeepWalk node representation has better performance under different similarity calculation methods. The performance of the link prediction model based on Cosine similarity is the best. With the increase of step size, AUC and Precision values are first rapidly improved and then remain unchanged.

**Fig 3:**
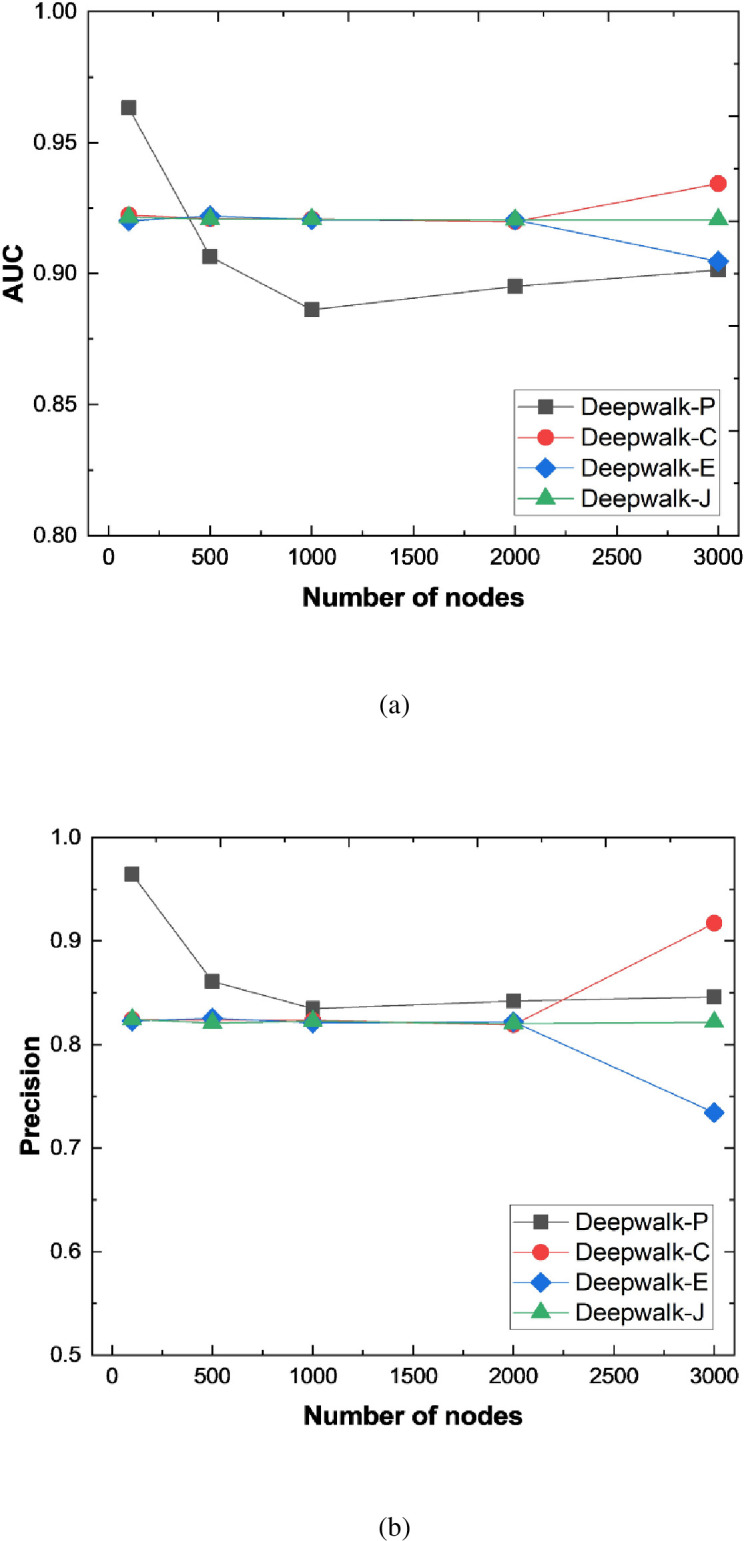
Performance of link prediction model corresponding to different number of nodes.

In addition, we further analyze the link prediction performance of the model under different node sizes, as shown in Fig 3. The abscissa is the number of nodes, and the ordinate is the selected evaluation index.

The experimental results show that the prediction performance of the model fluctuates to some extent as the node size increases. However, a comprehensive evaluation of the prediction performance shows that as the node size changes, the link prediction model based on Cosine similarity has better performance, and its AUC value and Precision value can almost remain unchanged. In addition, as the number of nodes increases, its performance also shows a rising trend.

**Fig 4:**
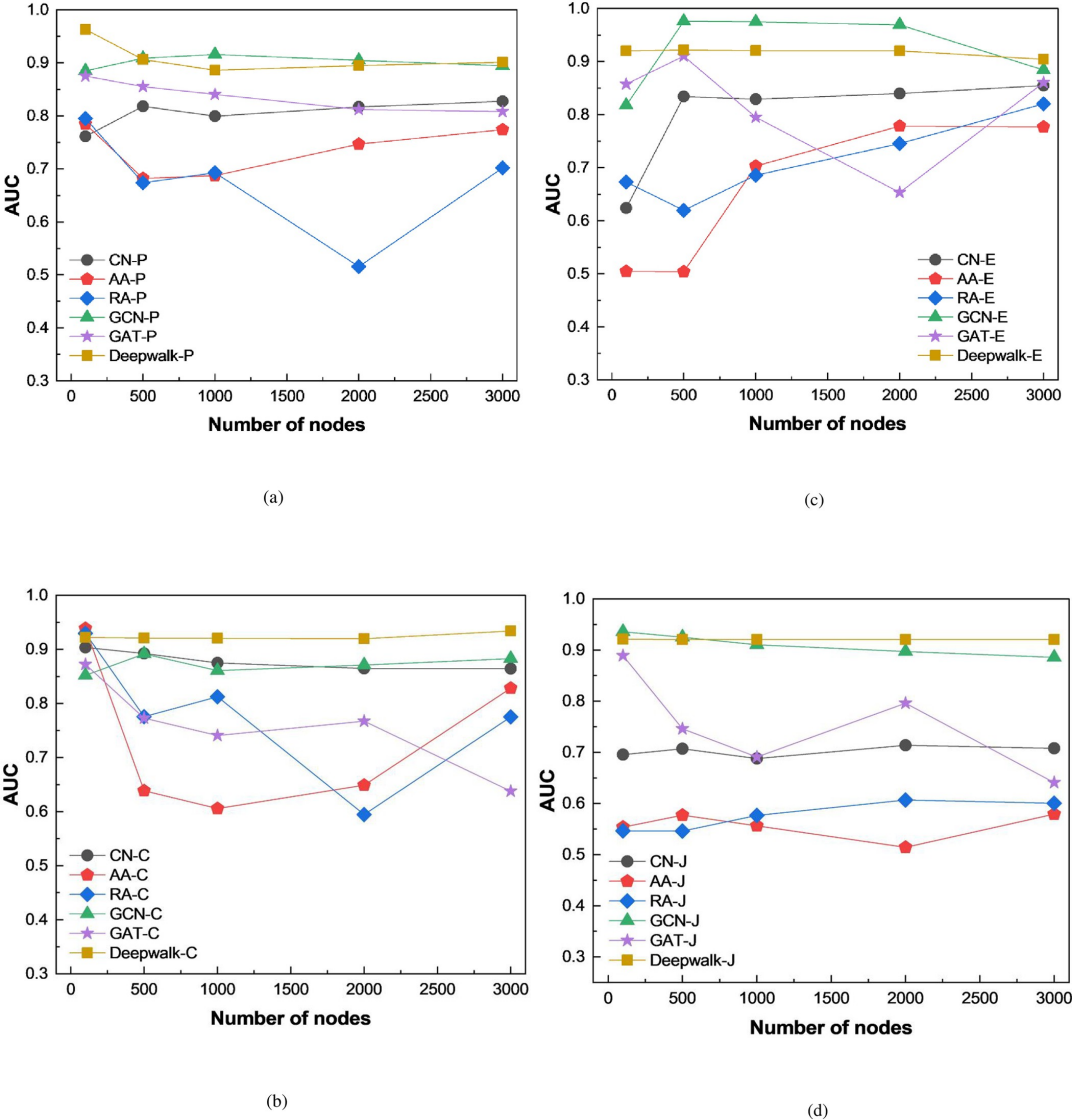
AUC of different link prediction algorithms.

**Fig 5:**
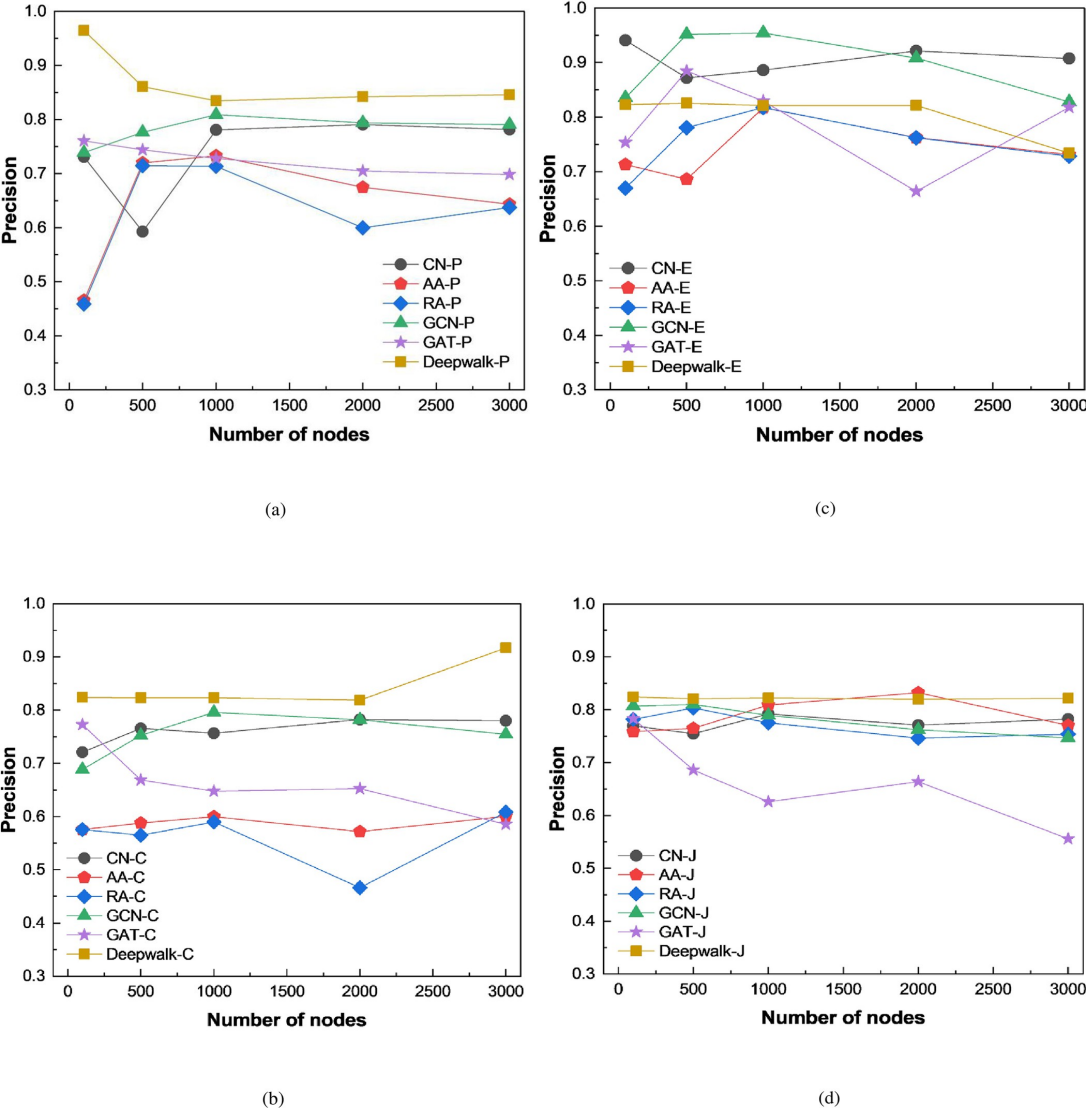
Precision of different link prediction algorithms.

In order to evaluate the effectiveness of the link prediction method proposed in this paper, it is compared with the performance of CN, AA, and RA link prediction algorithms. In addition, this paper also conducts comparative experiments by using GCN and GAT models to explore the results obtained by different feature extraction methods. The experimental results are shown in Figs 4 and 5.

The experimental results show that the link prediction method proposed in this paper is superior to the other three link prediction algorithms. Moreover, it can be found that link prediction methods based on graph neural networks can achieve good prediction results. And the prediction performance based on the DeepWalk model used in this study is generally superior to the GCN and GAT models.

By analyzing the principles of the used prediction models, it can be found that the main reason is that traditional link prediction algorithms can only directly predict based on the similarity relationships between nodes. This type of link prediction algorithms that consider the local similarities of nodes mainly utilize the topological structure information of the graph for link prediction, and can only use a small amount of network topological structure information. The utilization of graph information and node feature information is very limited, resulting in lower accuracy of prediction.

By comparison, deep learning-based link prediction algorithms are capable of mining high-dimensional information in graphs through their effective node representation methods, and hence can improve their prediction performance.

However, after analyzing the experimental results, it can be found that the prediction method proposed in this paper has a reduced prediction performance on the dataset obtained based on Euclidean similarity. From Table 1, it can be observed that the dataset obtained based on Euclidean similarity has lower density and fewer edges.

In addition, by comparing the above experimental results, we find that the performance of link prediction on the graph based on Cosine similarity is better than that of other similarity calculation methods. In order to explore the cause of this result, we compare the graph structure information brought by different similarity calculation methods in Table 1, and find that the graph network based on Cosine similarity calculation method has higher degree and density. This is because we set a link threshold between nodes. If the calculated similarity value is lower than the given threshold value, it will be removed.

It is noteworthy that the idea of the node representation method based on DeepWalk is first to use random walk algorithm to sample the sequence of nodes. Then it embeds and learns node representations based on this sequence. When the sampled network data is dense, that is, the number of edges is large, more sample data can be collected for representation learning training, so the final link prediction model has a better prediction performance. In order to verify the proposed conclusions, we take the data obtained from the Pearson similarity calculation as an example, and conduct comparative experiments on the prediction performance of models under different thresholds. Table 2 gives the network structure information under different thresholds, where *T*_*i*_ denotes different thresholds.

**Table 2:** Graph structure information under different link thresholds.

Method	Structure information	T_1_ = 0.4	T_2_ = 0.5	T_3_ = 0.6	T_4_ = 0.7	T_5_ = 0.8
Pearson	Average Degree	1919.35	1445.26	1066.31	578.29	294.2
	Density	0.64	0.48	0.36	0.19	0.1

**Fig 6:**
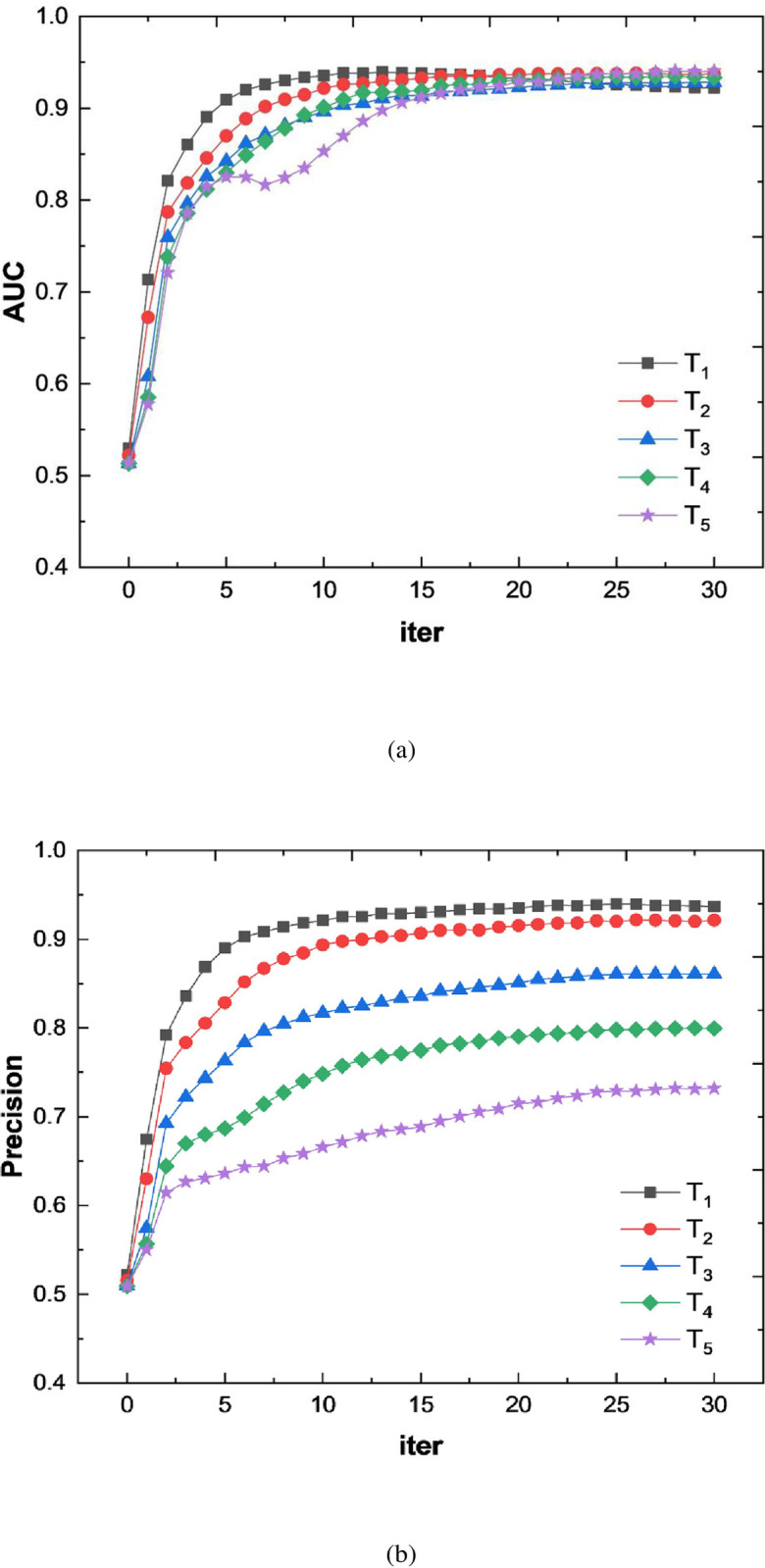
Performance of models under different link thresholds.

Fig 6 shows the corresponding experimental results. For the manufacturing service resource graph data obtained based on the Pearson similarity calculation method, as the link threshold increases during the construction of the graph dataset, the AUC and Precision values show an overall downward trend, thus verifying the above conclusions. Therefore, when constructing a cloud manufacturing service resource graph dataset, the predictive performance of the link prediction model can be improved by reducing the link threshold between nodes.

## Conclusion

In this paper, we proposed a CMfg service resource recommendation method based on graph neural network. We first constructed the manufacturing service resources on the CMfg service platform as graph datasets based on different similarity calculation methods. Then we used DeepWalk to learn the representations of nodes. On this basis, the manufacturing service resources corresponding to users’ transaction records and browsing records were constructed as subgraphs. And these representations were brought into the trained link prediction model to predict the possible links between manufacturing service resources, which can be used to recommend the corresponding manufacturing services to users based on the prediction results. This study also verified the proposed approach by using real-world cloud manufacturing data.

The experimental results shown that the link prediction model based on graph neural network can achieve better prediction performance, and its AUC value and Precision value can reach more than 90%. Its performance is also better than that of the other link prediction models for different node numbers. In addition, the experimental results also shown that the performance of the model can be gradually improved with the increase of graph density. Therefore, in practice, managers of cloud manufacturing service platforms should try their best to preserve the connections between resource nodes and set lower connection thresholds when processing the collected manufacturing resource data in order to achieve better recommendation results. We anticipate that the results of our study can contribute to enriching the recommending methods of cloud manufacturing service resources. And it can help alleviate the information overloading problem faced by users on cloud manufacturing service platforms.

However, there are some shortcomings in this study. For example, in reality, users’ manufacturing service demands may be diverse and complex, and hence how to carry out the combination of manufacturing service resource recommendation is undoubtedly key future research.

## Supporting information

S1 File (DOCX)
