# Supplementary material for: Research on cloud manufacturing service recommendation based on graph neural network
Source: PLoS One. 2023 Sep 26;18(9):e0291721. doi: 10.1371/journal.pone.0291721 (PMC10522046; doi:10.1371/journal.pone.0291721)
Supplement: S1 File — (DOCX) [file pone.0291721.s001.docx]

Data availability statement:

The data that support the findings of this study are openly available at [https://github.com/JUJUXIA-PYTHON/data.git].
